# Influence of Bacterial Physiology on Processing of Selenite, Biogenesis of Nanomaterials and Their Thermodynamic Stability

**DOI:** 10.3390/molecules24142532

**Published:** 2019-07-11

**Authors:** Elena Piacenza, Alessandro Presentato, Marta Bardelli, Silvia Lampis, Giovanni Vallini, Raymond J. Turner

**Affiliations:** 1 Microbial Biochemistry Laboratory, Department of Biological Sciences, University of Calgary, Calgary, AB T2N 1N4, Canada; 2 Environmental Microbiology and Microbial Biotechnology Laboratory, Department of Biotechnology, University of Verona, 37134 Verona, Italy

**Keywords:** biogenic nanomaterials, selenium nanomaterials, selenite, selenium nanoparticles, selenium nanorods, *Ochrobactrum*, thermodynamic stability, electrosteric stabilization

## Abstract

We explored how *Ochrobactrum* sp. MPV1 can convert up to 2.5 mM selenite within 120 h, surviving the challenge posed by high oxyanion concentrations. The data show that thiol-based biotic chemical reaction(s) occur upon bacterial exposure to low selenite concentrations, whereas enzymatic systems account for oxyanion removal when 2 mM oxyanion is exceeded. The selenite bioprocessing produces selenium nanomaterials, whose size and morphology depend on the bacterial physiology. Selenium nanoparticles were always produced by MPV1 cells, featuring an average diameter ranging between 90 and 140 nm, which we conclude constitutes the thermodynamic stability range for these nanostructures. Alternatively, selenium nanorods were observed for bacterial cells exposed to high selenite concentration or under controlled metabolism. Biogenic nanomaterials were enclosed by an organic material in part composed of amphiphilic biomolecules, which could form nanosized structures independently. Bacterial physiology influences the surface charge characterizing the organic material, suggesting its diverse biomolecular composition and its involvement in the tuning of the nanomaterial morphology. Finally, the organic material is in thermodynamic equilibrium with nanomaterials and responsible for their electrosteric stabilization, as changes in the temperature slightly influence the stability of biogenic compared to chemogenic nanomaterials.

## 1. Introduction

The role of microorganisms in the biogeochemical cycle of selenium (Se) has been established [1], although the vast genetic diversity of bacteria makes it difficult to fully elucidate the biological mechanisms behind the biochemistry of one of the most abundant and toxic Se species: the oxyanion selenite (SeO_3_^2−^) [2]. Since the beginning of the 20th century, a variety of microorganisms have been described for their ability to grow in the presence of Se oxyanions and bioprocess them into the less bioavailable elemental form (Se^0^) [3]. In the 1970s, this phenomenon started to be linked to the microbial capability of biosynthesizing Se nanostructures (SeNSs) with defined size and shape [4]. The simultaneous development of the nanotechnology field in terms of new synthetic procedures, nanomaterial (NM) characterization, and potential applications [5] resulted in an increased scientific focus on the possibility of using microorganisms as green and inexpensive catalysts to produce SeNSs [6], reaching its peak in the last 20 years in terms of dedicated research, whose interest was more devoted to investigating the mechanisms behind Se oxyanion bioprocessing than studying potential optimization of NM biosynthesis processes. To date, (1) Painter-type reactions involving thiol (RSH) groups [7,8,9], (2) enzymatic reduction by periplasmic or cytosolic oxidoreductases [10,11,12,13,14,15,16], (3) inorganic reactions with microbial metabolites [17], and (4) redox reactions mediated by siderophores [18] are the four strategies mainly acknowledged as able to achieve microbial processing of SeO_3_^2−^. However, no common mechanism has yet been identified for Se oxyanion biotransformation in bacteria, which instead depends more on the bacterial species investigated as well as the diverse physiological state of microorganisms.

The inherent complexity of bacteria also complicates the design of processes to produce SeNSs as controllable and predictable as chemogenic NSs, highlighting the necessity to study the mechanism of their biosynthesis. The choice of the organism to be used as the microbial cell factory, metal(loid) precursor concentration, pH, temperature, bacterial incubation timeframe, cell physiology, and localization of the precursor reduction events are parameters that must be considered when studying the biogenic production of SeNSs [19,20,21], as variations in these conditions can determine the physical-chemical characteristics. Among these features, morphology and size are crucial factors for NM applications [5], as they directly affect several fundamental properties of material on the nanoscale (e.g., electrical and optical features, potential toxicity or cellular uptake for medical applications) [5,22,23]. A key aspect of biogenically synthesized SeNSs is the presence of an organic material derived from the bacterial systems used, which seems to confer a naturally high degree of thermodynamic stability toward these NMs [24,25]. The function(s) and the composition of this organic material, as well as its variation upon changes in bacterial growth conditions, are not completely understood yet, constituting a black hole in the microbial nanotechnology field.

In the present study, we explored how the environmental isolate *Ochrobactrum* sp. MPV1 can tolerate high concentrations of SeO_3_^2−^. This strain has been previously described for its ability to biosynthesize Se nanoparticles (NPs) and nanorods (NRs) through SeO_3_^2−^ bioconversion [26,27]; thus, it was investigated for the removal of different SeO_3_^2-^ loads under different conditions to better understand the biomolecular process(es) behind this biotransformation. Metabolically controlled growth conditions were subsequently used to optimize the tuning of SeNS morphology previously observed [27], and all the recovered biogenic NSs were characterized, focusing on size and shape variations. Finally, the new insights presented in this study regarding composition, physical-chemical features, and role of the organic material enclosing SeNSs recovered from MPV1 cells revealed its paramount importance for the thermodynamic stabilization of biogenic NMs, making their coating with stabilizing agents typically required to prevent the aggregation of those chemically produced unnecessary.

## 2. Results

### 2.1. SeO_3_^2−^ Bioprocessing by MPV1 Cultures

**Figure 1:**
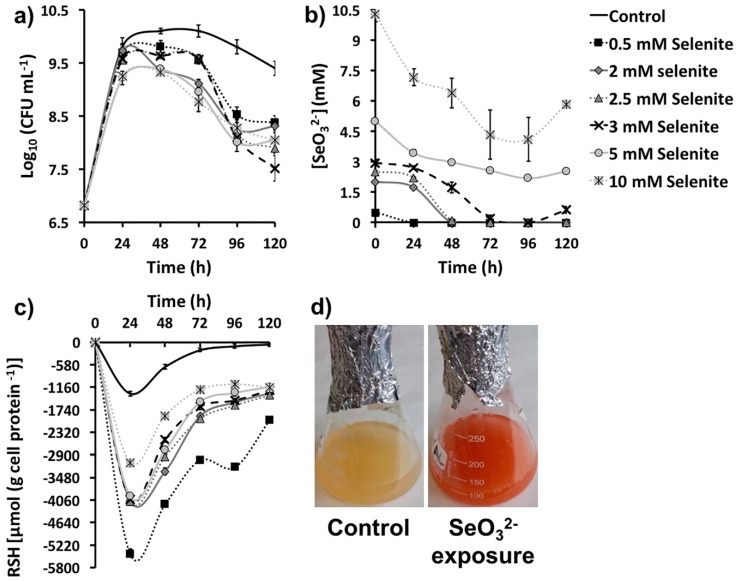
(**a**) Growth profiles, (**b**) SeO_3_^2−^ bioconversion, and (**c**) thiol (RSH) oxidation of MPV1 cultures grown in LB medium, or LB supplied with increasing concentrations (0.5, 2, 2.5, 3, 5, and 10 mM) of SeO_3_^2−^. In (**d**) is shown the bacterial culture color change upon cell exposure to selenite precursor.

The environmental isolate *Ochrobactrum* sp. MPV1 was previously described for its high tolerance to SeO_3_^2-^ exposure [26]. The ability of MPV1 to thrive under SeO_3_^2−^ toxicity was assessed by monitoring the bacterial growth and oxyanion removal under optimal conditions. The presence of increasing oxyanion concentrations (0.5, 2, 2.5, 3, 5, and 10 mM) did not strongly affect MPV1 growth in LB medium, even though a death phase was observed from 72 and 48 h onward upon exposure to 0.5–3 mM and 5–10 mM SeO_3_^2−^, respectively (Figure 1a). A general lower biomass production (ca. 1 log) was detected upon Se oxyanion addition compared with SeO_3_^2−^ free cultures, although any significant difference was not observed between the number of colony forming units (CFU) mL^−1^ at the latest time point considered (120 h) under oxyanion exposure (Figure 1a).

A complete removal of SeO_3_^2−^ was observed in the case of MPV1 cultures exposed to 0.5, 2 and 2.5 mM SeO_3_^2−^ after 24, 48, and 72 h of growth respectively, whereas higher oxyanion concentrations (i.e., 3, 5, or 10 mM) were only partially bioprocessed (ca. 2.5 mM) within 120 or 168 h, as indicated by the reappearance over the time of a certain amount of oxyanion in the growth medium (Figure 1b, Table 1). Thus, regardless of the initial oxyanion concentration supplied, 2.5 mM SeO_3_^2−^ appeared to be the threshold value for oxyanion biotic removal for MPV1 cells under these experimental conditions.

The oxidation of RSH pools in MPV1 cultures under SeO_3_^2−^ pressure mimicked the trend for bacterial cells not exposed to Se oxyanions, reaching the maximum extent after 24 h of growth (Figure 1c). Overall, the pressure exerted by SeO_3_^2−^ on MPV1 cells led to a greater loss of reduced RSH with respect to SeO_3_^2−^ free cultures. The highest amount of oxidized RSH was measured for MPV1 cells exposed to 0.5 mM SeO_3_^2−^, whereas a similar level of loss of reduced RSH was detected upon bacterial incubation with oxyanion concentrations ranging from 2 to 5 mM (Figure 1c). The lowest extent of RSH oxidation was measured during growth in the presence of 10 mM SeO_3_^2−^ (Figure 1c), although similar oxyanion removal rates were observed for MPV1 cells exposed to 2.5–10 mM SeO_3_^2−^ (Figure 1b and Table 1). The amount of oxidized RSH after 24 h of bacterial growth and the initial SeO_3_^2−^ concentration supplied were inversely and linearly related (Figure S1), suggesting that not only RSH chemistry was involved in the oxyanion bioprocessing.

### 2.2. Characterization of Se Nanostructures Produced by MPV1 Cells

Subsequent to MPV1 growth in the presence of SeO_3_^2−^, biogenic extracts containing SeNSs were recovered and characterized from a physical-chemical perspective. Table 2 lists the biogenic extracts, their acronyms (used hereinafter), and the procedure applied for their recovery.

**Figure 2:**
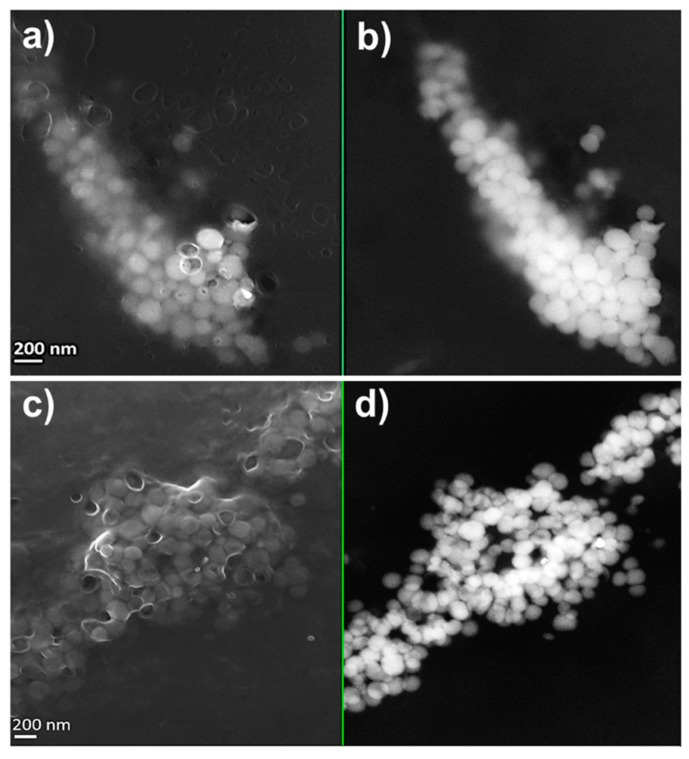
Scanning electron micrographs of (**a**,**b**) SeNPs_MPV1-0.5_24_e_ and (**c**,**d**) SeNPs_MPV1-0.5_120_e_ recovered from MPV1 cells grown for 24 h: (**a**) InLens detector and (**b**) backscattered electron detector, or 120 h: (**c**) InLens detector and (**d**) backscattered electron detector in the presence of 0.5 mM SeO_3_^2−^.

**Figure 3:**
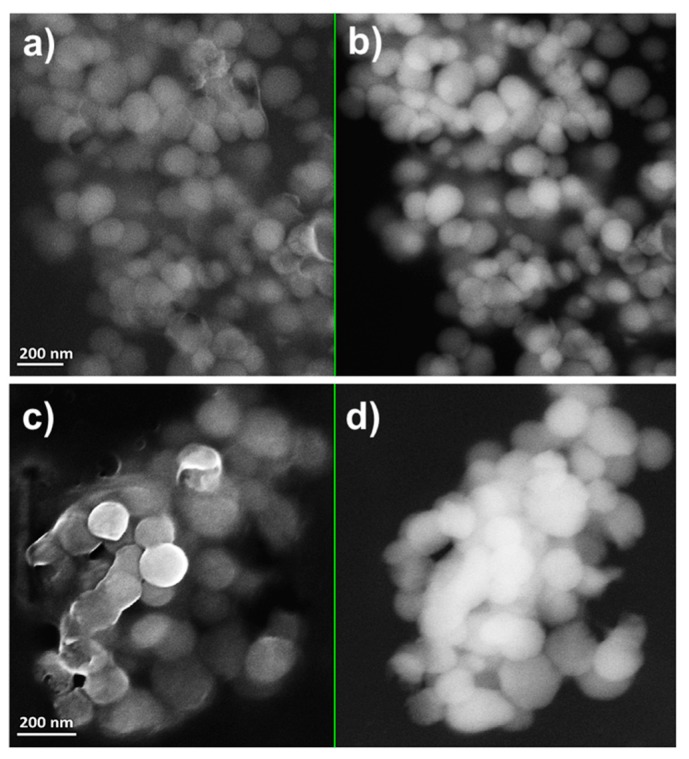
Scanning electron micrographs of (**a**,**b**) SeNPs_MPV1-2_48_e_ and (**c**,**d**) SeNPs_MPV1-2_120_e_ recovered from MPV1 cells grown for 48 h: (**a**) InLens detector and (**b**) backscattered electron detector, or 120 h: (**c**) InLens detector and (**d**) backscattered electron detector in the presence of 2 mM SeO_3_^2−^.

MPV1 cells biosynthesized both SeNPs and SeNRs as a function of the initial SeO_3_^2−^ bioconversion. TEM observations revealed the production of SeNPs when MPV1 was grown in LB medium supplied with 0.5–5 mM SeO_3_^2−^ (Figure S2a–c), whereas NPs and few NRs were detected in the biogenic extracts upon bacterial growth in the presence of 10 mM SeO_3_^2−^ (Figure S2d,d1). Regardless of the oxyanion concentration tested, a slightly electron-dense material was observed in all the biogenic extracts analyzed, in which structures having electron patterns resembling those of lipid-like vesicles were identified (Figure S2(a1)). Bigger and non-uniform electron-dense SeNPs were detected in SeNPs_MPV1-0.5_120_e_ and SeNPs_MPV1-2_120_e_ compared with SeNPs_MPV1-5_120_e_ and SeNPs_MPV1-10_120_e_ (Figure S2), which indicated a possible agglomeration of small NPs upon MPV1 incubation with low SeO_3_^2−^ concentrations (i.e., 0.5 and 2 mM). To assess whether this phenomenon is attributable to the fast bioprocessing of 0.5 or 2 mM Se oxyanion performed by MPV1 cells (Figure 1b), and a consequent high number of intracellular Se atoms available for NS formation, SEM imaging (Figure 2 and Figure 3) was performed on SeNPs_MPV1-0.5_120_e_ and SeNPs_MPV1-2_120_e_, enabling a comparison with SeNPs_MPV1-0.5_24_e_ and SeNPs_MPV1-2_48_e_, which were recovered concomitantly with the disappearance of SeO_3_^2−^ from the cell-free spent medium (Figure 1b).

As a result, these extracts contained significantly smaller SeNPs (Figure 2 and Figure 3) compared to those imaged by TEM (Figure S2a,a1,b), which were closely associated with each other due to the presence of an enclosing matrix composed of light elements, but retaining NP identity. Any significant difference was not detected in the average diameter of SeNPs synthesized during the early (24 or 48 h) or late (120 h) stage of MPV1 growth in the presence of 0.5 and 2 mM SeO_3_^2−^, which ranged between 120 and 150 nm (Table 3).

**Figure 4:**
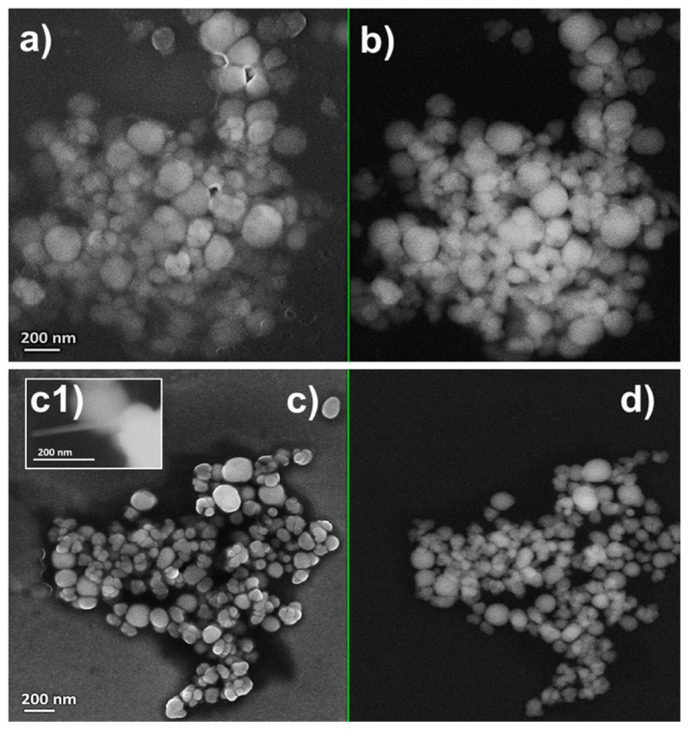
Scanning electron micrographs of (**a**,**b**) SeNPs_MPV1-5_120_e_ and (**c**,**d**) SeNPs_MPV1-10_120_e_ recovered from MPV1 cells grown for 120 h in the presence of 5 mM ((**a**) InLens detector and (**b**) backscattered electron detector) or 10 mM SeO_3_^2-^ mM ((**c**) InLens detector and (**d**) backscattered electron detector).

Similar range of sizes were also obtained for NPs present within SeNPs_MPV1-5_120_e_ and SeNPs_MPV1-10_120_e_ (Figure 4 and Table 3), underlining that this could be a potential and natural stability range for biogenic SeNP diameters produced by MPV1. Finally, the presence of few SeNRs in the extracts recovered upon MPV1 growth in the presence to the highest SeO_3_^2-^ concentration tested (10 mM) was further confirmed by SEM imaging (Figure 4c1).

### 2.3. Tuning of Se Nanostructure Morphology by Varying MPV1 Physiological State

**Figure 5:**
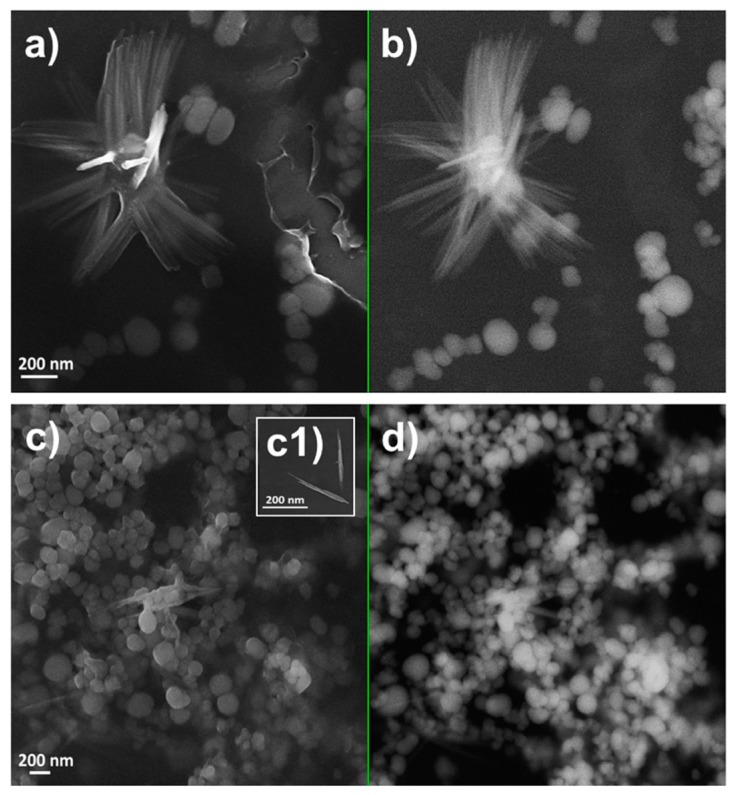
Scanning electron micrographs of (**a**,**b**) SeNSs_MPV1_G_e_ and (**c**,**d**) SeNSs_MPV1_P_e_ recovered from MPV1 cells grown for 120 h in the presence of glucose and 0.5 mM SeO_3_^2-^ ((**a**) InLens detector and (**b**) backscattered electron detector) or pyruvate and 0.5 mM SeO_3_^2-^ ((**c**) InLens detector and (**d**) backscattered electron detector).

Since MPV1 showed its proficiency in biosynthesizing a mixed population of SeNPs and SeNRs under metabolically controlled growth conditions [27], the effect of a pre-culturing step on cell adaptation was explored in an attempt to further tune SeNS production. The first observations revealed that the change in the pre-culturing conditions led to higher bioprocessing of Se oxyanions (ca. 0.3 mM) by MPV1 cells within 120 h of incubation (Figure S3) with respect to what was previously reported [27]. Secondly, the increased SeO_3_^2−^ removal was accompanied by the biosynthesis of mixed populations of SeNPs and SeNRs regardless of the carbon source supplied (Figure 5), as opposed to previous observations where only glucose-grown cells produced SeNRs [27]. Particularly, SeNSs_MPV1_G_e_ contained mostly SeNRs (Figure 5a,b), but a lower number of NRs was detected compared to NPs within SeNSs_MPV1_P_e_ (Figure 5c,c1,d). SeNPs present in both the biogenic extracts were ca. 130 nm in size, comparable to those recovered from MPV1 cells grown in the rich LB medium, whereas longer SeNRs were produced by cells oxidizing glucose instead of pyruvate as the only sources of carbon and energy (Table 3). SEM observations also indicated the presence of a material enclosing the biogenic SeNSs and preventing their aggregation (Figure 5), which resembled the SEM images obtained for the extracts recovered from MPV1 grown in LB medium (Figure 2, Figure 3 and Figure 4).

### 2.4. Physical-Chemical Characterization of the Biogenic Se Nanostructure Extracts

Since the biogenic NSs investigated were enclosed in some sort of matrix likely arising from MPV1 cells, EDX spectroscopy was performed on the extracts to evaluate their elemental composition. Except for the silicon (Si; K_α_ = 1.739 KeV) signal due to the mounting of the biogenic extracts onto Si wafers, all the EDX spectra collected showed the presence of two Se peaks (K_α_ = 11.207 KeV and L_α_ = 1.379 KeV), one signal for carbon (C; K_α_ = 0.277 KeV), oxygen (O; K_α_ = 0.525 KeV), and sulfur (S; K_α_ = 2.307 KeV), whereas the presence of nitrogen (N; K_α_ = 0.392 KeV) was detected only in the extracts recovered from MPV1 cells grown in LB medium (Table 4). Thus, the elemental composition of biogenic SeNS extracts displayed the occurrence of elements typical of biomolecules constituting bacterial cells (i.e., nucleic acids, proteins, lipids, and carbohydrates), suggesting the organic nature of the material enclosing the biogenic NSs, whose complexity and element distribution is shown in Figure S4.

DLS analyses were subsequently performed to study the associated organic material recovered from the biogenic SeNS extracts, revealing its ability to auto-assemble in the nanorange with size distributions between 130 and 170 nm (Table 5), being comparable to the average diameter of biogenic SeNPs calculated from SEM imaging (Table 3). This organic material was also characterized as having a reasonable level of monodispersity according to the evaluated PdI index (<0.3). This implies that the recovered organic material can auto-assemble into structures that are comparable in size (Table 5), potentially mediated by the amphiphilic molecules produced by the bacteria.

ζ analyses showed that the organic material had a negative surface charge similar to that measured for the whole biogenic NS extracts, which ranged from −22 to −16 mV (Table 6).

Combining these observations, it is tempting to propose that this organic material dictates the size, shape, and charge of the SeNSs. The only biogenic extracts and recovered organic material that displayed ζ values close to neutrality were those isolated from MPV1 cells grown under metabolically controlled conditions (Table 6), suggesting further a possible difference in composition between the analyzed extracts.

### 2.5. Role of Organic Material in Thermodynamic Stabilization of Biogenic Se Nanostructures

A first attempt to evaluate the nature of the interaction between SeNSs and the organic material enclosing them was conducted by performing several washing steps aimed at obtaining this material free from NSs. The removal of the organic material led to the irreversible aggregation of the NSs themselves. Since this phenomenon was observed for all the SeNS extracts analyzed, the organic material surrounding these NSs seemed not to be covalently attached to the surface of the nanomaterial core, but most likely reversibly adsorbed on their surfaces, as well as present in solution in thermodynamic equilibrium, which was perturbed after each washing step, as previously suggested by Presentato et al. [28]. These results indicate the key role played by organic material in the colloidal stability of biogenic SeNSs, preventing their aggregation.

**Figure 6:**
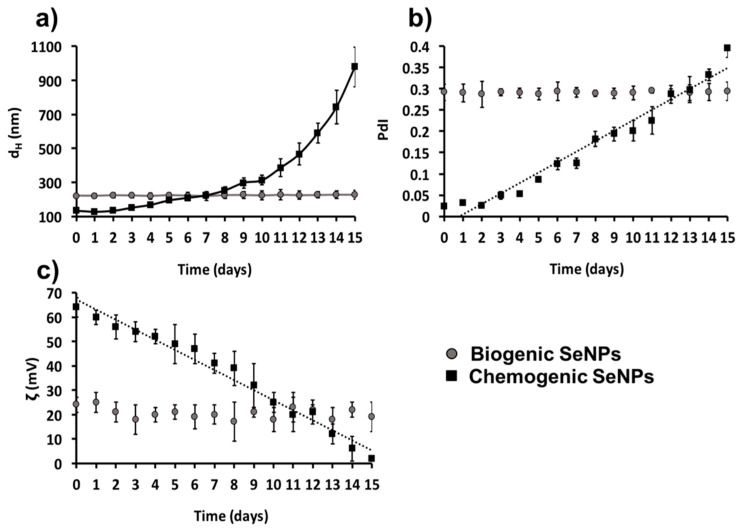
(**a**) Evaluation of the hydrodynamic diameter (d_H_), (**b**) polydispersity index (PdI) values, and (**c**) zeta potential (ζ) absolute values of SeNPs_MPV1-0.5_e_ and l-cys SeNPs incubated for 15 days at 27 °C. Linear relationships were observed between either (**b**) PdI or (**c**) ζ values of l-cys SeNPs and the incubation times (*R^2^* = 0.96 and 0.97, respectively).

Considering the similarity in composition and behavior of all the biogenic samples highlighted by EDX, DLS, and ζ analyses, we studied the thermodynamic stability of SeNS extracts on SeNPs_MPV1-0.5_120_e_ incubated for 15 days at room temperature, using l-cys SeNPs for chemogenic comparison. As a result, SeNPs_MPV1-0.5_120_e_ maintained high thermodynamic stability in suspension over the timeframe considered, showing only slight variations in size distribution, surface charge, and PdI value within 15 days (Figure 6), the latter being always below the threshold value (<0.3). Conversely, l-cys SeNPs were strongly affected by this treatment, reaching a complete instability from day 7 onward, as indicated by the exponential increase in the d_H_ (Figure 6a) and the PdI value (Figure 6b), as well as the decrease in the absolute ζ value, which was almost neutral at the latest stage of incubation (2 ± 1 mV; Figure 6c). The formation of black precipitates in solution further confirmed the higher thermodynamic instability and polydispersity of l-cys-SeNPs compared to biogenic NSs.

## 3. Discussion

The investigation conducted to unveil potential mechanism(s) exploited by MPV1 to cope with increasing concentrations of SeO_3_^2−^ (0.5–10 mM) highlighted the growth and oxyanion removal rates (Figure 1a,b, Table 1) comparable to those described for most SeO_3_^2−^ tolerant bacteria [3,29,30,31,32,33,34,35]. Since Se oxyanions exceeding 2.5 mM reappeared in the growth medium upon exposure to 3, 5, and 10 mM SeO_3_^2−^ (Figure 1b, Table 1), 2.5 mM SeO_3_^2−^ appears to be the threshold concentration biotically processed by MPV1 cells under these experimental conditions, as also observed in the case of *Moraxella bovis* [36]. This evidence indicates that the bioprocess of SeO_3_^2−^ by MPV1 might involve: (1) the uptake of increasing concentrations Se oxyanions, (2) their bioaccumulation and bioconversion up to 2.5 mM, and (3) a gradual release of exceeding SeO_3_^2−^ amounts. This last step could be due to either cell lysis events, however unlikely, as similar death events were observed in bacterial cultures incubated with all SeO_3_^2−^ concentrations (Figure 1a), or a saturation of the cellular systems responsible for SeO_3_^2−^ removal, which led to the release of oxyanions to reach a sort of equilibrium between the intra- and extra-cellular environments [34].

The high level of RSH oxidation measured in MPV1 cells exposed to 0.5 mM SeO_3_^2−^ (Figure 1c) indicates a major involvement of these reactive groups for SeO_3_^2−^ removal. Other cellular systems (i.e., enzymes) seemed to be involved in the bioprocessing of SeO_3_^2−^ concentrations exceeding 0.5 mM, as suggested by (1) the ability of MPV1 cells to biotically remove ca. 2.5 from 10 mM SeO_3_^2−^ after 168 h of incubation (Figure 1b, Table 1), and yet (2) the RSH levels recover toward later incubation times defining a low level of sustained oxidized RSHs (Figure 1c), and (3) their minor contribution to the oxyanion conversion as function of SeO_3_^2−^ concentration, as depicted by the linear relationship observed in Figure S1. The presence of an inhibitor for glutathione (GSH) synthesis, *S-n*-butyl homocysteine sulfoximine (BSO), only slightly affected the biotic removal of 2 mM SeO_3_^2−^, revealing only a six-hour delay in the process [26]. Thus, the key role played by GSHs in MPV1 cells is to bioconvert Se oxyanions, yet ancillary enzymatic mechanism(s) can be induced as function of SeO_3_^2−^ concentration and time of exposure. Ubiquitous enzymes, like NAD(P)H-dependent thioredoxin reductases and flavin oxidoreductases, sulfate or sulfite reductases, or fumarate reductases, were identified as responsible for the biotic reduction of high concentrations (from 2 to 10 mM) of SeO_3_^2−^ [30,32,33,34,35,37]. In this regard, NADPH-dependent reduction activity toward high concentrations (5 mM) of SeO_3_^2−^ was found in the cytoplasmic and, to a minor extent, in the periplasmic fractions of MPV1 cells [26]. SeO_3_^2−^ bioprocessing can also be mediated by intracellular SeO_3_^2−^ reductases [34,38], lignin peroxidase [39], chromate (CrsF), ferric (FerB) and arsenate reductases (ArsH) [37], or the metalloid-selective channel porin ExtI [40]. Thus, enzymatic systems might be accountable for the bioconversion of high oxyanion concentrations in MPV1, whereas low amounts of SeO_3_^2−^ are likely bioprocessed through Painter-type reactions.

Regardless of the initial concentration of SeO_3_^2−^ precursor, MPV1 biosynthesized SeNPs as the main product of Se oxyanion bioconversion (Figure 2, Figure 3, Figure 4 and Figure S2). The process behind the formation of NSs relies on a number of parameters (i.e., precursor concentration, reducing agent, reaction time, the concentration of elemental atoms) that influence the rate of growth, morphology, and size of NMs [41,42]. Due to the complexity of a biological system, the type of cell factory and the localization of precursor reduction events must be accounted for by NS biosynthesis, as they directly influence the concentration of metal atoms available for NM formation. Previous reports showed that the reduction of SeO_3_^2−^ occurred in the cytoplasm of MPV1 [26,27], leading to the confinement of many Se atoms in the small cellular volume, increasing the chances to exceed the critical level of these atoms to form Se nuclei [43], which eventually grow as NPs. Thus, the MPV1 intracellular environment can improve the synthesis of SeNSs even at low concentration of Se atoms with respect to chemogenic procedures.

Overall, NMs synthesized by microorganisms generally feature high polydispersity in size [25], which mostly depends on the uneven distribution of the metal(loid) precursor within the cells during bacterial growth, resulting in the accumulation of different intracellular concentrations of elemental atoms, which can determine diverse NS production rates [43]. However, despite the different growth conditions tested, the average diameter of biogenic SeNPs was always between 90 and 140 nm (Table 3), indicating a good monodispersity in size, in line with most studies reported to date [44]. Although NPs are classically defined as particles having a diameter between 1 and 100 nm, the unique physical-chemical properties of these biogenic Se-structures [27] and the proximity of their size with the range in question allow them to be considered as NPs, accordingly to some of the definitions coined to date for these NMs [45,46]. The monodispersity of biogenic SeNPs may indicate their natural stability within this range size due to the existence of an organic material composed of biomolecules produced by bacterial cells that participate to control NP diameter [47,48]. The close association of SeNPs with the organic material was further supported by SEM imaging, which highlighted the presence of a matrix composed of light elements (Table 4) and enclosing SeNPs (Figure 2, Figure 3 and Figure 4). TEM micrographs revealed the occurrence clusters of NPs in SeNPs_MPV1-0.5_120_e_ and SeNPs_MPV1-2_120_e_ (Figure S2a,b), likely caused by the high bioprocessing rate of low SeO_3_^2−^ concentrations. Since any significant difference was not observed in the growth profile of MPV1 cells upon exposure to diverse oxyanion concentrations (Figure 1a), the bacterial incubation with 0.5 and 2 mM SeO_3_^2−^ corresponded to the highest precursor (SeO_3_^2−^)-to-reducing agent (RSHs and enzymatic systems) ratio, which mediated the fastest oxyanion bioprocessing observed (Figure 1b and Table 1). This would result in the buildup of a high concentration of Se atoms over a short period of time, causing the rapid formation of SeNPs and their eventual agglomeration [49,50] in the intracellular environment, even though their complete aggregation was prevented by the presence of the organic material. The low extent of oxyanion bioprocessing under MPV1 exposure to either 5 or 10 mM (Figure 1b, Table 1) led to a decreased amount of Se atoms available for NP synthesis over the time period [20], preventing the detection of big clusters within TEM micrographs (Figure S2c,d).

**Figure 7:**
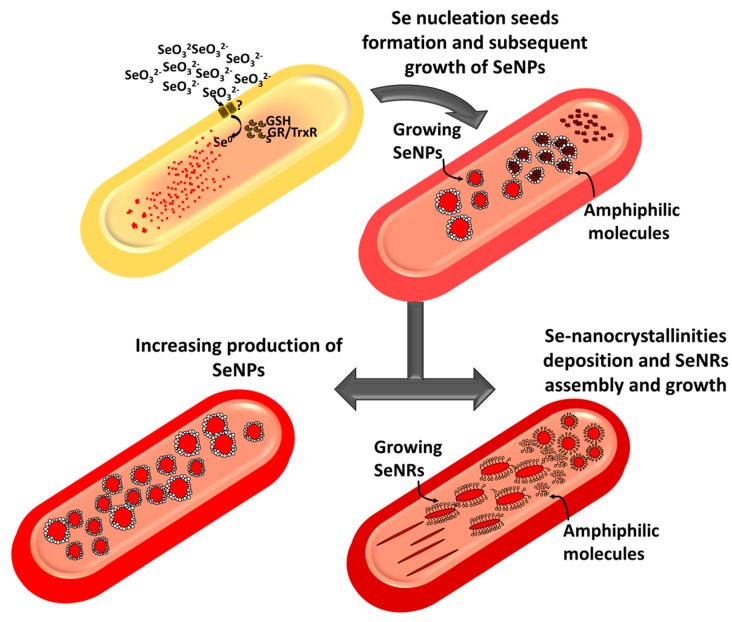
Fast SeO_3_^2−^ uptake is followed by its rapid bioconversion within MPV1 cells, causing a build-up of Se^0^ atoms in the intracellular environment. Consequently, Se atoms eventually aggregate with each other, and once the solubility threshold concentration is reached, they form intracellular nucleation seeds. This event is followed by the generation of a high amount of intracellular SeNPs, which can be mediated by amphiphilic biomolecules present within the cells that can also provide thermodynamic stability to the forming NSs. SeNRs production is instead favored by the exposure of MPV1 to high concentrations of SeO_3_^2−^ as well as its growth-eliciting specific metabolisms, most likely due to the co-production of a high amount of amphiphilic biomolecules as stress response that can act as surfactants, providing a template for the growth of Se nucleation seeds along one axis.

Previous studies concerning the characterization of biogenic SeNSs showed the existence of an organic material playing a key role in their synthesis and stabilization [6,44]. Over the past few years, FTIR spectroscopy has been the most-used technique to assess the presence of biomolecules associated with SeNSs, enabling the detection of proteins, carbohydrates, and lipids within most of the extracts analyzed [20,32,37,47,51,52,53,54,55,56,57], including those recovered from MPV1 cells grown under optimal conditions [26]. Here, the detection of light elements attributable to biomolecules co-produced by the bacterial strain alongside Se (Table 4) highlighted a certain degree of variability among the biogenic NSs, likely due to the exploitation of multiple strategies by MPV1 to remove Se oxyanions [20,32]. The detection of N in some cases might be ascribed to the occurrence of proteins or metabolites within the biogenic extracts [26], whereas the constant presence of S signal may be due to the involvement of RSHs in SeO_3_^2−^ bioprocessing for MPV1 cultures [20,21,58]. The narrow size distributions of the organic material (Table 5) suggested that it mostly contained amphiphilic biomolecules able to form nanosized aggregates (e.g., micelles and vesicles) when suspended in aqueous solution [28,59]. The low PdI values indicated the ability of these biomolecules to form monodisperse structures [60]. Since Se does not have a net charge in its elemental state (Se^0^), the negative ζ values (Table 6) may indicate that negatively charged biomolecules were part of the biogenic extracts, whose charges can be attributed to the presence of either carboxyl (–COO^−^) or phosphate (–PO_4_^2−^) functional groups [28,61]. Although similar in elemental composition, the biogenic extracts recovered from MPV1 cells grown under metabolically controlled conditions showed ζ values closed to neutrality (Table 6), potentially indicating differences in terms of biomolecular composition, depending on the metabolism exploited by MPV1 to cope with Se oxyanion toxicity. The different bacterial physiological states determined morphological changes of SeNSs (Figure 5), resulting in the production of both NPs and NRs, also observed in the case of *Shewanella* sp. HN-41 [19], *Lysinibacillus* sp. ZYM-1 [20], and *Rhodococcus aetherivorans* BCP1 [28]. This phenomenon can be ascribed to the bivalent nature of Se, as once amorphous NPs are formed, they can spontaneously dissolve and release Se atoms [62], which might precipitate as nanocrystallinites and grow in one direction to attain a more thermodynamic stable state, allowing NRs to form [63]. This process is favored by the co-presence of amphiphilic molecules (e.g., surfactants having a bulky structure) that can act as templates to guide the deposition of Se atoms and their growth in one direction [64]. In this regard, the synthesis of biosurfactants was earlier reported for *Ochrobactrum* genus bacterial strains when grown under stress conditions [65], whereas the shift from SeNPs to SeNRs was previously observed in MPV1-glucose grown cells [27]. Here, this change in NS morphology was emphasized due to the different MPV1 pre-culturing conditions, and cells also thriving under pyruvate and SeO_3_^2−^ pressure-produced SeNRs (Figure 5c,d), suggesting a direct influence of the bacterial physiology on the biosynthesis of different nanomaterial morphologies. Based on both the evidence collected here and previous studies [26,27], a putative mechanism illustrating SeO_3_^2−^ bioprocessing and SeNS production by MPV1 is proposed in Figure 7.

The biomolecules present in the extracts are also responsible for the thermodynamic stability of biogenic SeNSs, as indicated by the formation of insoluble Se precipitates upon physical removal of the organic material. This conclusion was further supported by the slight effect of the temperature on both surface charge and d_H_ of SeNPs_MPV1-0.5_120_e_, as opposed to l-cys SeNPs (Figure 6), whose electrostatic stabilization was completely lost within 15 days. This phenomenon may be due to the overall development of electrostatic (charged moieties) and steric (bulky amphiphilic molecules) interactions between the organic material and the SeNSs within the biogenic extracts, generating the electrosteric stabilization effect [25,28,52,61], which is used to strongly stabilize chemogenic NMs [64].

## 4. Materials and Methods

### 4.1. Bacterial Culture Conditions

*Ochrobactrum* sp. MPV1, isolated from a dump site for roasted pyrites at a former sulfuric acid production plant [26], was pre-cultured for 16 h at 27 °C with shaking (200 rpm) in 13-mL test tubes containing 5 mL of Luria Bertani (LB) medium composed of sodium chloride (NaCl; 10 g L^−1^), tryptone (10 g L^−1^), and yeast extract (5 g L^−1^). The cells were then inoculated (1% *v*/*v*) and cultured under microaerophilic conditions for 120 h at 27 °C with shaking (200 rpm) in fresh LB medium with 0.5, 2, 2.5, 3, 5, or 10 mM of sodium selenite (Na_2_SeO_3_). For MPV1 cells cultured under metabolically controlled conditions, the cells were pre-cultured in defined medium (DM) [66] supplied with either glucose or pyruvate (0.5% *w*/*v*) as the sole carbon and energy source, then inoculated (1% *v*/*v*) in fresh pre-culturing medium with the addition of 0.5 mM Na_2_SeO_3_.

The bacterial growth profile was evaluated every 24 h using the spot plate count method, with the data reported as the logarithm of the CFU per milliliter (log_10_(CFU mL^−1^)) for each biological replica (*n* = 3) with SD.

All the reagents used were purchased from Sigma-Aldrich^®^ (Milan, Italy) and were all analytical grade.

### 4.2. Biotic SeO_3_^2-^ Removal Efficiency

SeO_3_^2-^ bioprocessing by MPV1 cells was determined following the protocol described by Kessi et al. [67], evaluating the oxyanion residual concentration present in the cell-free spent medium every 24 h of growth by measuring the absorbance of the selenium-2,3-diaminonaphthalene complex at 377 nm, using a 1-cm path length quartz cuvette (Hellma^®^, Milan, Italy) and a Varian Cary^®^ 50 Bio UV-Vis Spectrophotometer (Agilent Technologies, Milan, Italy). Calibration curve (*R^2^* = 0.99) was determined using 0, 50, 100, 150, and 200 nmol of SeO_3_^2−^. The residual SeO_3_^2−^ concentrations (mM) are reported as average value (*n* = 3) with SD.

### 4.3. Measurement of Thiol Oxidation as Consequence of SeO_3_^2−^ Bioprocessing

Thiol (RSH) oxidation was monitored by sampling MPV1 cultures every 24 h of growth supplied with increasing SeO_3_^2−^ concentrations, following the procedure established by Turner et al. [68]. The absorbance of the suspension containing oxidized RSHs was read at 412 nm using a 1-cm path length Acrylic cuvette (Sarstedt, Verona, Italy) and a Varian Cary^®^ (Agilent Technologies, Milan, Italy) 50 Bio UV–Vis Spectrophotometer. RSH concentration was determined by using the known extinction coefficient of 5,5-dithio-bis-2-nitrobenzoic acid (DTNB; 1.36 × 10^4^ M^−1^ cm^−1^), and normalizing the data over the total amount of cell proteins, which were collected from MPV1 cultures after 48 h of growth and quantified using a modified Lowry assay [69]. The concentration of RSH estimated at the beginning of cell incubation (t_0_ = 0 h) was subtracted to RSH contents evaluated over the timeframe considered to report the data as loss of reduced RSH from the original pool (*n* = 3) with SD.

### 4.4. Preparation and Recovery of Biogenic Se Nanomaterial Extracts and Their Supernatants

The biogenic SeNSs (i.e., NPs or NRs) were recovered using the optimized protocols described by Piacenza et al. and Presentato et al. [27,28]. Briefly, MPV1 biomass was centrifuged (3000 ×*g* for 20 min) and resuspended in 10 mL of 1.5 mM Tris-HCl (Sigma-Aldrich^®^) buffer (pH 7). The cells were then disrupted by means of ultrasonication (UP50H Hielscher) at 50 W for 5 min (30 s of burst interspersed by 30 s of pause on ice). The cell debris was removed by centrifugation (3000 ×*g* for 20 min), whereas the supernatant containing SeNPs was filtered using 0.20 μm Filtropur (Sarstedt). To collect the biogenic SeNPs, the filtered solution was centrifuged (20,000 ×*g* for 30 min), forming the nanoparticle pellet resuspended in sterile distilled water. The solution containing SeNRs was treated with 1-Octanol in a ratio of 1:4 to remove excess cell debris, avoiding the filtering step that might alter nanorod integrity. Since there was a close but not covalent association between the SeNSs and the surrounding organic material, the latter was recovered according to Presentato et al. [28]. Specifically, the extracts were centrifuged (20,000 ×*g* for 30 min) to pellet down SeNSs, and the supernatant, now containing the organic material striped off from the biogenic NMs, was the subject of further physical-chemical characterization.

### 4.5. Physical-Chemical Characterization of Biogenic Se Nanomaterial Extracts

TEM was performed by depositing 5 µL of biogenic extracts onto carbon coated copper grids (CF300-CU, Electron Microscopy Sciences, Rome, Italy), which were then air-dried prior their imaging by means of a Philips CM100 TEM (Milan, Italy) operating at 80 kV. Similarly, 5 µL of each extract were deposited onto Crystalline Silicon wafers (type N/Phos, size 100 mm, University WAFER, Milan, Italy) mounted on Specimen Aluminum stubs (TED PELLA, INC., Milan, Italy), air-dried and visualized using a Zeiss Sigma VP field emission scanning electron microscope (FESEM, Milan, Italy), which was coupled with a Bruker XFlash R 4 detector (Milan, Italy) to acquire energy dispersive X-ray (EDX) spectra. FESEM micrographs were subsequently analyzed using ImageJ software (1.50i, National Institutes of Health, Rockville Pike Bethesda, MD, USA) to calculate the average size (i.e., diameter and length) of SeNPs or NRs by measuring 100 randomly chosen SeNSs for each biogenic extract considered.

Dynamic light scattering (DLS) and Zeta potential (ζ) measurements were performed at pH = 7 and 25 °C on 1 mL solutions of biogenic SeNS extracts and the recovered organic material enclosing SeNSs by means of a Zen 3600 Zetasizer Nano ZS™ from Malvern Instruments (Milan, Italy) using spectrophotometric cuvettes (10 × 10 × 45 mm Acrylic Cuvettes, Sarstedt, Verona, Italy) and folded capillary Zeta cells (Malvern Instruments, Milan, Italy), respectively.

### 4.6. Monitoring Thermodynamic Stability of Biogenic Se Nanomaterial Extracts and Chemogenic Se Nanoparticles

The chemogenic procedure described by Li et al. was used to synthesize l-cysteine SeNPs (l-cys SeNPs) by mixing l-cysteine (50 mM) and Na_2_SeO_3_ (100 mM) at a ratio 4:1 at room temperature [49] to obtain NPs ranging in size between 150 and 200 nm, similar to that of SeNPs_MPV1-0.5_24_e_. The thermodynamic stability of l-cys SeNPs and SeNPs_MPV1-0.5_24_e_ was evaluated in terms of hydrodynamic diameter (d_H_), polydispersity index (PdI), and ζ changes by incubating these nanomaterials at room temperature (25 °C) and pH 7 over a period of 15 days.

## 5. Conclusions

*Ochrobactrum* sp. MPV1 showed high resilience to SeO_3_^2−^ toxicity, indicating the existence of multiple intracellular systems (i.e., RSHs and enzymatic systems) that may be responsible for removing up to 2.5 mM SeO_3_^2−^, which is the threshold concentration of oxyanion processing by this bacterial strain. As a consequence of Se oxyanion bioconversion, MPV1 produced SeNSs, whose morphology was dependent on either the SeO_3_^2−^ concentration supplied or the bacterial physiological state, leading to the biosynthesis of NPs or NRs. Particularly, we highlighted the existence of a stability range for SeNP diameter, and improved the knowledge regarding the production and the physical-chemical properties of SeNSs by MPV1, focusing on the role of the organic material enclosing the NSs, which is of utmost importance for the development of electrosteric interactions mediating the thermodynamic stability of biogenic SeNSs as opposed to those of chemical synthesis.

## Figures and Tables

**Table 1:** SeO_3_^2−^ bioprocessing efficacy of MPV1 cultures grown in LB medium over time.

	SeO_3_^2-^ Removal (mM) as Function of Its Initial Concentration					
Time (h)	0.5	2	2.5	3	5	10
24	0.5	0.27 ± 0.09	0.31 ± 0.02	0.30 ± 0.08	1.56 ± 0.13	2.83 ± 0.12
48	-	2	2.39 ± 0.04	1.28 ± 0.11	2.02 ± 0.05	3.62 ± 0.09
72	-	-	2.5	2.79 ± 0.13	2.43 ± 0.04	5.67 ± 0.05
96	-	-	-	3	2.81 ± 0.03	5.89 ± 0.07
120	-	-	-	2.47 ± 0.10	2.46 ± 0.09	4.16 ± 0.04
144	N.D.	N.D.	N.D.	N.D.	2.51 ± 0.10	2.93 ± 0.09
168	N.D.	N.D.	N.D.	N.D.	2.54 ± 0.08	2.51 ± 0.11

Note: - represents the complete removal of the initial SeO_3_^2−^ concentration supplied to the growth medium; N.D. stands for Not Determined.

**Table 2:** Conditions used to produce biogenic SeNS extracts, their acronyms and procedures used for their recovery from MPV1 cells.

MPV1 Culture Conditions to Produce SeNSs	Acronym	Recovery Procedure
Growth for 24 h in the presence of 0.5 mM SeO_3_^2−^	SeNPs_MPV1-0.5_24_e_	[27]
Growth for 120 h in the presence of 0.5 mM SeO_3_^2−^	SeNPs_MPV1-0.5_120_e_	
Growth for 48 h in the presence of 2 mM SeO_3_^2−^	SeNPs_MPV1-2_48_e_	
Growth for 120 h in the presence of 2 mM SeO_3_^2−^	SeNPs_MPV1-2_120_e_	
Growth for 120 h in the presence of 5 mM SeO_3_^2−^	SeNPs_MPV1-5_120_e_	
Growth for 120 h in the presence of 10 mM SeO_3_^2−^	SeNSs_MPV1-10_120_e_	
Growth for 120 h in the presence of glucose and 0.5 mM SeO_3_^2−^	SeNSs_MPV1_G_e_	[28]
Growth for 120 h in the presence of pyruvate and 0.5 mM SeO_3_^2−^	SeNSs_MPV1_P_e_	

**Table 3:** Average diameter or length of SeNPs or SeNRs produced by MPV1 cells under different growth conditions.

Biogenic SeNS Extracts	Average NP Diameter (nm)	Average NR Length (nm)
SeNPs_MPV1-0.5_24_e_	122 ± 40	N.D.
SeNPs_MPV1-0.5_120_e_	146 ± 25	N.D.
SeNPs_MPV1-2_48_e_	118 ± 36	N.D.
SeNPs_MPV1-2_120_e_	132 ± 21	N.D.
SeNPs_MPV1-5_120_e_	125 ± 32	N.D.
SeNPs_MPV1-10_120_e_	92 ± 26	N.D.
SeNSs_MPV1-G_e_	125 ± 37	513 ± 92
SeNSs_MPV1-P_e_	127 ± 52	418 ± 115

**Table 4:** Elemental composition of biogenic SeNS extracts obtained through EDX spectroscopy.

Biogenic Extract	Se	C	O	N	S
SeNPs_MPV1-0.5_120_e_	✓	✓	✓	✓	✓
SeNPs_MPV1-2_120_e_	✓	✓	✓	✓	✓
SeNPs_MPV1-5_120_e_	✓	✓	✓	✓	✓
SeNPs_MPV1-10_120_e_	✓	✓	✓	✓	✓
SeNSs_MPV1-G_e_	✓	✓	✓	-	✓
SeNSs_MPV1-P_e_	✓	✓	✓	-	✓

✓ indicates the presence of the element in the extracts; - represents the absence of the element in the extracts.

**Table 5:** Hydrodynamic diameter (d_H_) and PdI values of the organic material removed from biogenic SeNSs.

Organic Material Samples	d_H_ (nm)	PdI
OM_SeNPs_MPV1-0.5_120_e_	140 ± 23	0.135
OM_SeNPs_MPV1-2_120_e_	131 ± 13	0.173
OM_SeNPs_MPV1-5_120_e_	155 ± 20	0.167
OM_SeNPs_MPV1-10_120_e_	167 ± 35	0.181
OM_SeNSs_MPV1-G_e_	143 ± 17	0.110
OM_SeNSs_MPV1-P_e_	152 ± 23	0.105

Note: OM_ represents the organic material removed from the NSs contained within the indicated extract.

**Table 6:** Surface charge (ζ) of biogenic SeNS extracts and their supernatants removed from NSs.

Biogenic Extracts	ζ (mV)	Organic Material Samples	ζ (mV)
SeNPs_MPV1-0.5_120_e_	−18 ± 1	OM_SeNPs_MPV1-0.5_120_e_	−18 ± 3
SeNPs_MPV1-2_120_e_	−21 ± 2	OM_SeNPs_MPV1-2_120_e_	−13 ± 4
SeNPs_MPV1-5_120_e_	−22 ± 1	OM_SeNPs_MPV1-5_120_e_	−19 ± 2
SeNPs_MPV1-10_120_e_	−16 ± 3	OM_SeNPs_MPV1-10_120_e_	−12 ± 4
SeNSs_MPV1-G_e_	−2 ± 2	OM_SeNSs_MPV1-G_e_	−6 ± 5
SeNSs_MPV1-P_e_	3 ± 1	OM_SeNSs_MPV1-P_e_	4 ± 2

Note: OM_ represents the organic material removed from the NSs contained within the indicated extract.

## Supplementary Materials

The following are available online at https://www.mdpi.com/1420-3049/24/14/2532/s1, Figure S1: Linear relationship (*R^2^* = 0.97) between the concentration of SeO_3_^2−^ supplied to MPV1 cultures in LB medium and that of oxidized thiols (RSHs) detected after 24 h of growth, Figure S2: Transmission electron micrographs of (**a**) SeNPs_MPV1-0.5_120_e_, (**b**) SeNPs_MPV1-2_120_e_, (**c**) SeNPs_MPV1-5_120_e_, and (**d**) SeNSs_MPV1-10_120_e_ recovered from MPV1 cells grown for 120 h in the presence of (**a**) 0.5, (**b**) 2, (**c**) 5, or (d) 10 mM SeO_3_^2−^. Black arrows indicate the emergence of SeNPs or SeNRs and the electron-dense material surrounding them, whereas (**a1**) the inlet highlights the presence of lipid-like vesicles within the extract, Figure S3: SeO_3_^2−^ bioprocessing by MPV1 cells grown for 120 h in DM supplied with (**a**) glucose or (**b**) pyruvate and 0.5 mM SeO_3_^2−^, Figure S4: Elemental map obtained through Energy-Dispersive X-Ray (EDX) spectroscopy performed on SeNSs_MPV1-G_e_.
