# Supplementary figures and images for: Influence of Bacterial Physiology on Processing of Selenite, Biogenesis of Nanomaterials and Their Thermodynamic Stability
Source: Molecules. 2019 Jul 11;24(14):2532. doi: 10.3390/molecules24142532 (PMC6681009; doi:10.3390/molecules24142532)

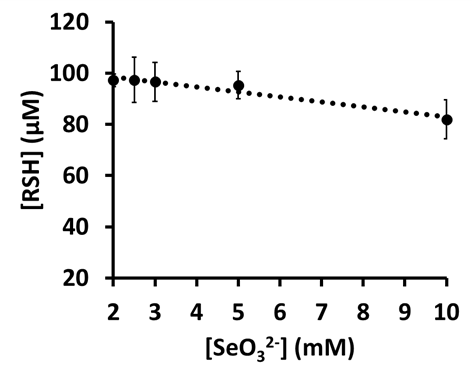

Supplement: Supplementary file 1 [file molecules-24-02532-s001.zip › Figure S1.png]

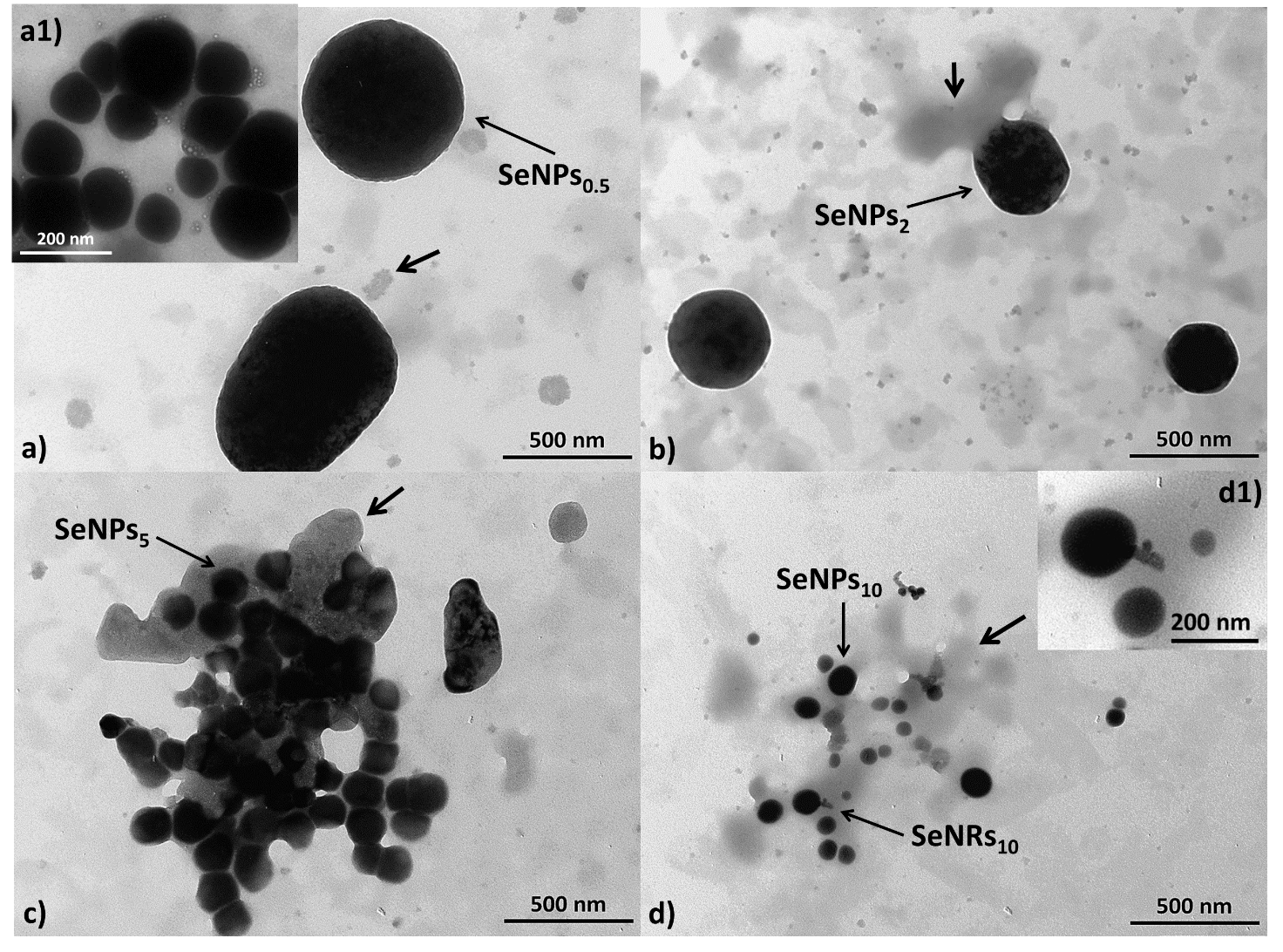

Supplement: Supplementary file 1 [file molecules-24-02532-s001.zip › Figure S2.png]

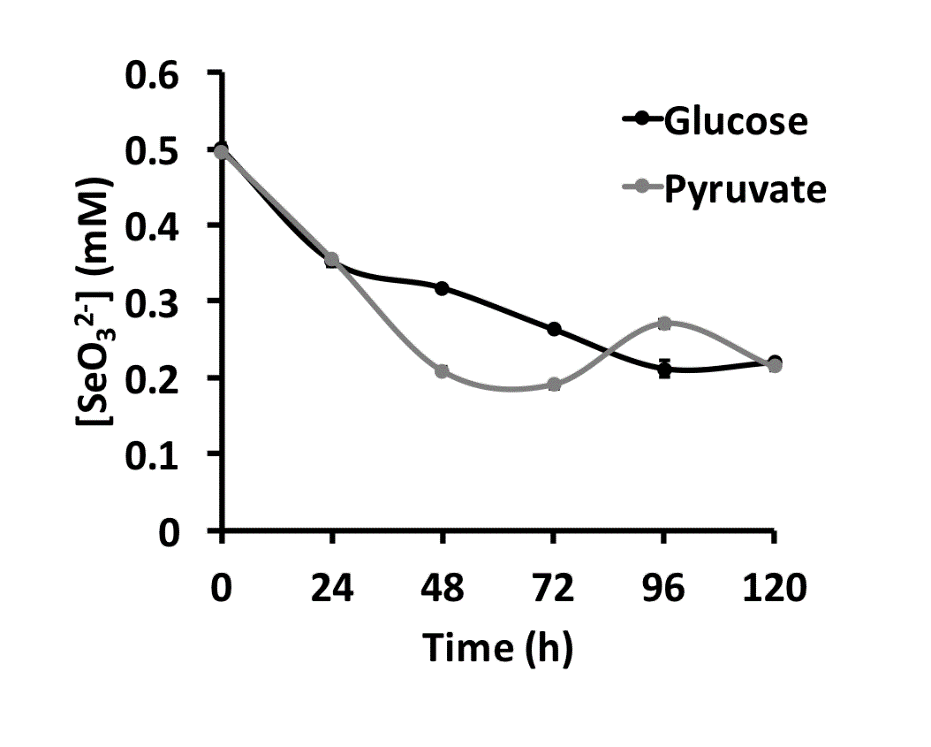

Supplement: Supplementary file 1 [file molecules-24-02532-s001.zip › Figure S3.png]

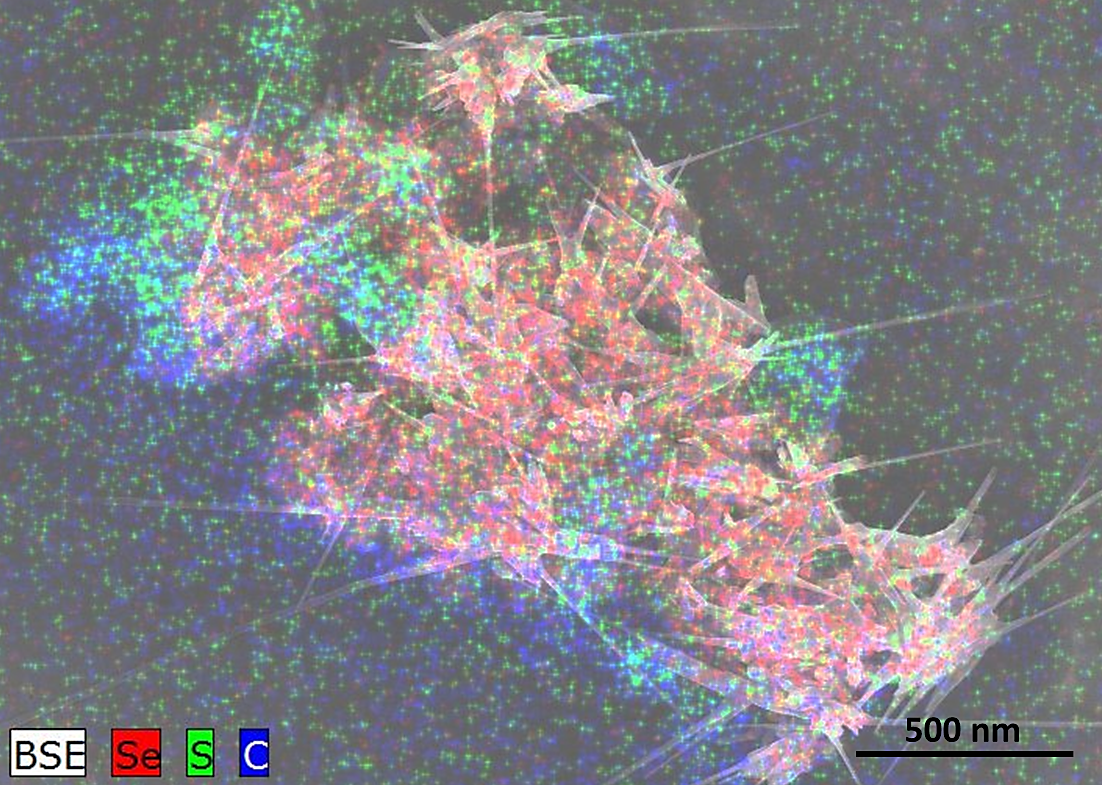

Supplement: Supplementary file 1 [file molecules-24-02532-s001.zip › Figure S4.png]
